# The potential protective role of Parkinson’s disease against hypothyroidism: co-localisation and bidirectional Mendelian randomization study

**DOI:** 10.3389/fnagi.2024.1377719

**Published:** 2024-05-14

**Authors:** Jiang Lei, Wenxuan He, Yao Liu, Qinxin Zhang, Yingyao Liu, Qican Ou, Xianli Wu, Fenglin Li, Jiajia Liao, Yousheng Xiao

**Affiliations:** ^1^Department of Neurology, The First Affiliated Hospital of Guangxi Medical University, Nanning, China; ^2^Department of Neurology, The First People’s Hospital of Nanning, Nanning, China; ^3^Department of Rehabilitation Medicine, Jiangbin Hospital of Guangxi Zhuang Autonomous Region, Nanning, China

**Keywords:** Parkinson’s disease, Mendelian randomization, single-nucleotide polymorphism, hypothyroidism, co-localization

## Abstract

**Background:**

The association between hypothyroidism and Parkinson’s disease (PD) has sparked intense debate in the medical community due to conflicting study results. A better understanding of this association is crucial because of its potential implications for both pathogenesis and treatment strategies.

**Methods:**

To elucidate this complex relationship, we used Bayesian co-localisation (COLOC) and bidirectional Mendelian randomization (MR) analysis. COLOC was first used to determine whether hypothyroidism and PD share a common genetic basis. Subsequently, genetic variants served as instrumental variables in a bidirectional MR to explore causal interactions between these conditions.

**Results:**

COLOC analysis revealed no shared genetic variants between hypothyroidism and PD, with *a posteriori* probability of hypothesis 4 (PPH4) = 0.025. Furthermore, MR analysis indicated that hypothyroidism does not have a substantial causal effect on PD (OR = 0.990, 95% CI = 0.925, 1.060, *p* = 0.774). Conversely, PD appears to have a negative causal effect on hypothyroidism (OR = 0.776, 95% CI = 0.649, 0.928, *p* = 0.005).

**Conclusion:**

Our findings suggest the absence of shared genetic variants between hypothyroidism and PD. Interestingly, PD may inversely influence the risk of developing hypothyroidism, a finding that may inform future research and clinical approaches.

## 1 Introduction

Parkinson’s disease (PD) is the second most common neurodegenerative disease after Alzheimer’s disease, and its incidence continues to increase (Pavlou and Outeiro, 2017). The dyskinesia symptoms of PD–bradykinesia, tremor, rigidity and postural balance deficits–are the result of dopaminergic neuronal deficits and the accumulation of α-synuclein (Jakubowski and Labrie, 2017; Mohd Murshid et al., 2022). Dysregulation of dopamine metabolism, oxidative stress and neuroinflammation are key factors contributing to dopaminergic neuronal deficits (Dexter and Jenner, 2013; Labbé et al., 2016; Chang and Chen, 2020). Hypothyroidism, characterised by low thyroid hormone levels, is associated with oxidative stress and inflammatory responses (Mancini et al., 2016; de Carvalho et al., 2018). There is conflicting evidence about the relationship between hypothyroidism and the risk of PD. For example, a Danish study involving more than 80,000 people found no significant association between hypothyroidism and PD, whereas studies in Taiwan and South Korea have shown that patients with hypothyroidism have an increased risk of PD (Rugbjerg et al., 2009; Chen et al., 2020; Kim et al., 2021). Hypothyroidism is known to increase the risk of anaemia, obesity and hyperlipidaemia, all of which are associated with an increased risk of developing PD (Mohammadi et al., 2021). Given the mixed results of observational studies and potential confounding factors, it remains difficult to establish a causal relationship between hypothyroidism and PD.

Mendelian randomization (MR), which uses single-nucleotide polymorphisms (SNPs) as instrumental variables to estimate the causal effect of exposure on outcomes, can help researchers identify causal relationships between diseases and avoid the confounding problems often found in traditional studies (Liu et al., 2021). MR has proven to be a powerful and convenient tool for investigating risk factors associated with PD (Senkevich et al., 2023; Song et al., 2024). Bayesian co-localisation (COLOC) is a method that uses Bayesian statistical principles to assess whether two or more traits share the same genetic variation (Chen et al., 2024). This is important for understanding how different traits are linked by common biological pathways or mechanisms. Some studies have used MR in conjunction with COLOC, an approach that has revealed a common genetic basis between psoriasis and multiple sclerosis and helped to identify potential therapeutic targets for multiple sclerosis (Lin et al., 2023; Patrick et al., 2023).

Traditional observational studies often encounter difficulties in establishing a causal relationship between PD and hypothyroidism, the main obstacle being the exclusion of confounding factors. To overcome this limitation, MR offers a powerful alternative approach that is particularly effective in overcoming the limitations of traditional studies in making causal inferences. Our study uses a genome-wide association study (GWAS) dataset containing two diseases, which provides a good basis for using both COLOC and MR analysis methods. The combination of these two approaches could provide a solid basis for genetic commonality and causal inference to reveal genetic and causal links between hypothyroidism and PD, and provide new biological insights into these two potentially related disorders, thus opening up new avenues for clinical practice and future research directions.

## 2 Materials and methods

### 2.1 Data sources and study populations

We obtained data from published GWAS,^1^ focusing on datasets that provide comprehensive insights into PD and hypothyroidism (Table 1). As this study used publicly available data, ethical approval was not required as each dataset had already received ethical approval.

**Table 1:** Source of exposure and outcome GWAS data.

Phenotype	GAWSID	Sample size (cases/controls)	SNPs	Population
Parkinson’s disease	finngen_R10_G6_PARKINSON	4,681/407,500	19,345,634	European
Hypothyroidism	finngen_R10_E4_CONGEIOD	935/349,717	19,344,524	European

### 2.2 Colocalization analysis

The COLOC analysis was based on the following assumptions (Wallace, 2020). Independence assumption: associations between traits are caused directly by a common genetic variant and not by other indirect factors such as confounding variables. COLOC uses a Bayesian probability model to assess the strength of the evidence that two traits share the same SNPs. COLOC can derive five types of probability, including cases where the two traits are independent and have no common variant, each has its own variant, and one variant is shared. We used the hypothetical posterior probability 4 (PPH4) in the COLOC algorithm to assess whether the two share genetic variants, with PPH4 > 80% defined as the two sharing the same genetic variant as an influence (Deng et al., 2022). This analysis was performed using the “coloc” software package.^2^

### 2.3 MR study design

Our MR study design was based on three critical assumptions: (A) SNPs are significantly associated with exposure, serving as an instrumental variable; (B) these SNPs are not associated with known confounders; and (C) the effect of SNPs on outcome is entirely mediated by exposure factors.

### 2.4 Selection of instrumental variables

SNPs were used as instrumental variables according to the following four criteria: a. *p* < 5 × 10^8^; b. removal of chain imbalances (kb = 10,000 and r^2^ = 0.001) (Hu et al., 2023); c. search for these SNPs in PhenoScanner^3^ and removal of SNPs associated with confounders and outcomes (Hu et al., 2022); d. calculation and selection of SNPs with F-statistics >10, which can mitigate the effects of potential bias (Hu et al., 2023).

### 2.5 MR analysis

We chose inverse variance weighted (IVW), weighted median, MR-Egger and weighted mode for MR analyses, and considered *p* < 0.05 to be statistically significant (Han et al., 2022). Heterogeneity was assessed using MR-Egger and IVW regressions, with *p* > 0.05 indicating no heterogeneity. The presence of horizontal pleiotropy was assessed using the Egger intercept, with *p* > 0.05 indicating the absence of horizontal pleiotropy (Hu et al., 2022, 2023). Visualization results are presented as rejection-by-exclusion tests, forest plots, scatter plots and funnel plots. All statistical analyses were performed using the “TwoSampleMR” package in R (version 4.2.3).

## 3 Result

### 3.1 Co-localization between hypothyroidism and PD

**Figure 1:**
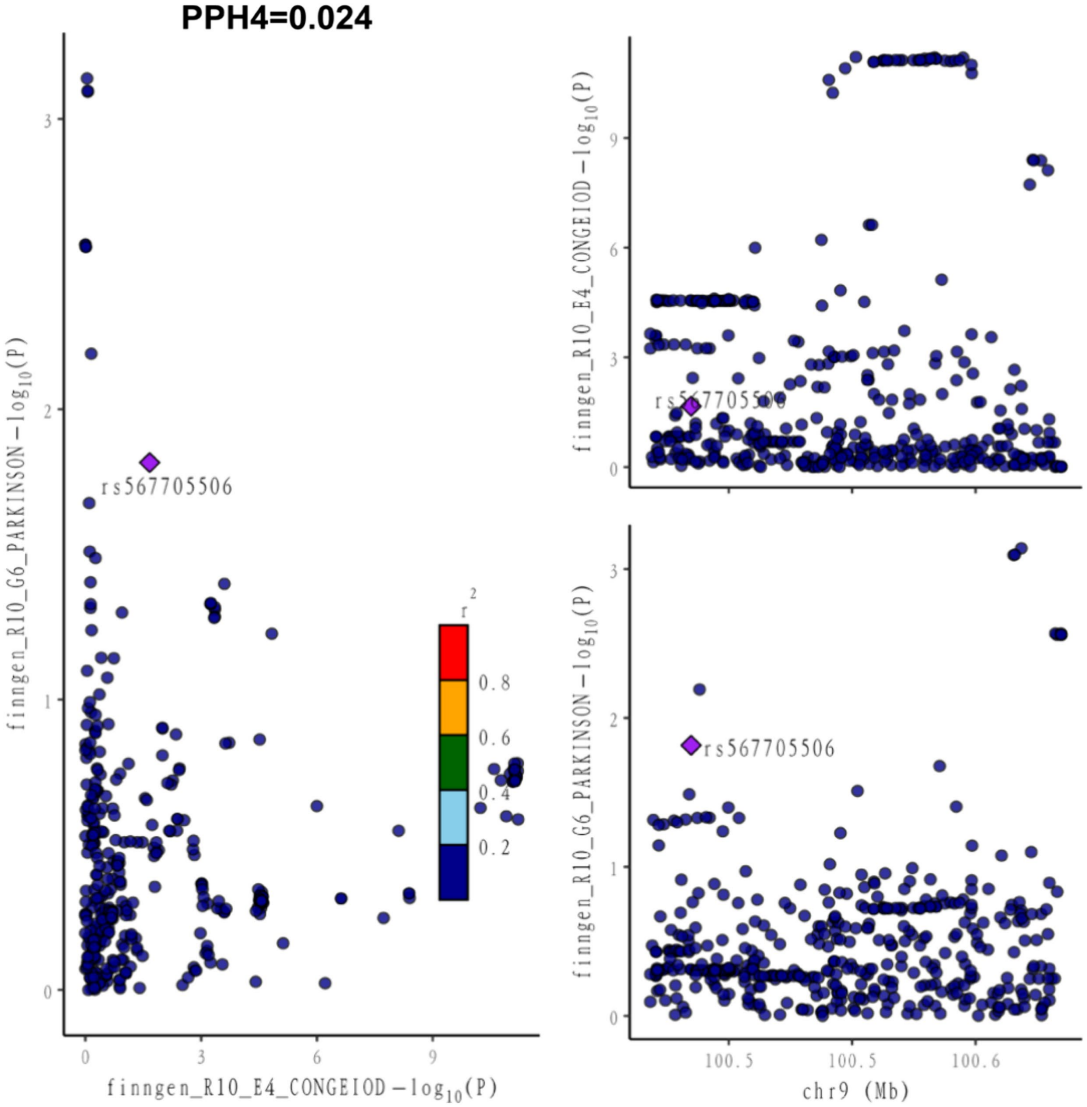
Co-localization between hypothyroidism and PD. PPH4 stands for posterior probability of hypothesis 4. PPH4 > 80% defined as both sharing the same genetic variant as an influencing factor.

In our paired GWAS analysis of hypothyroidism and PD, the COLOC PPH4 was below 80% (PPH4 = 0.024), suggesting that there is insufficient evidence to support that they share a common genetic locus (Figure 1). This finding suggests that there may not be a direct genetic link between hypothyroidism and PD, and that they may affect patients through different biological pathways.

### 3.2 For performing MR analysis SNPs

When selecting SNPs as instrumental variables from the GWAS dataset, we used methodological mention criteria to ensure that these SNPs were strongly associated with the phenotype, as detailed in Supplementary Table S1. This ensured the precision and reliability of our analysis.

### 3.3 MR analysis, sensitivity analysis and visualization

#### 3.3.1 Results of MR analysis of hypothyroidism on PD

Using a MR analysis with validated instrumental variables, the MR-IVW results showed no causal effect of hypothyroidism on PD (OR = 0.990, 95% CI = 0.925, 1.060, *p* = 0.774). The MR Egger (OR = 1. 006, 95% CI = 0.824, 1.227, *p* = 0.953), weighted median (OR = 0.968, 95% CI = 0.899, 1.042, *p* = 0.389) and weighted mode (OR = 1.034, 95% CI = 0.902, 1.185, *p* = 0.641) analyses were consistent with the MR-IVW analysis (Table 2). Although our analysis did not show a causal effect of hypothyroidism on PD. This result helps to rule out hypothyroidism as a potential risk factor or protective factor for PD. The Cochran’s Q statistic in the MR-Egger and IVW regressions showed heterogeneity in the Cochran’s Q test (*p* < 0.05) (Supplementary Table S2). Our horizontal multivariate test showed no horizontal multivariate *p* > 0.05 in the instrumental variables (Supplementary Table S3). Sensitivity analyses using the leave-one-out method confirmed the stability of our results (Supplementary Figure S1A). Forest, scatter and funnel plots supported the results of our MR analyses (Supplementary Figures S2A–C).

**Table 2:** Bidirectional MR analysis of the relationship between hypothyroidism and PD.

Exposures	Outcome	Method	SNPs	OR	95% CI	*p*
Hypothyroidism	PD	IVW	11	0.990	0. 925, 1.060	0.774
		MR Egger		1.006	0.824, 1.227	0.953
		Weighted median		0.968	0.899, 1.042	0.389
		Weighted mode		1.034	0.902, 1.185	0.641
PD	Hypothyroidism	IVW	20	0.776	0.649, 0.928	0.005
		MR Egger		0.866	0.618, 1.215	0.418
		Weighted median		0.829	0.649, 1.058	0.130
		Weighted mode		0.924	0.635, 1.343	0.682

#### 3.3.2 Results of MR analysis of PD on hypothyroidism

The same MR analysis method was used to infer the causal effect of PD on hypothyroidism. Interestingly, although the MR Egger (OR = 0.886, 95% CI = 0.618, 1.215, *p* = 0.418), weighted median (OR = 0.829 95% CI = 0.649, 1.058, *p* = 0.130) and weighted mode (OR = 0.682 95% CI = 0.635, 1.343, *p* = 0.682) analyses did not agree with the results of the MR-IVW analyses, the MR-IVW results suggested that PD reduced the risk of developing hypothyroidism (OR = 0.776 95% CI = 0.649, 0.928, *p* = 0.005). The Cochran’s Q statistic for the MR-Egger and IVW regressions suggested that the Cochran’s Q test was not heterogeneous (*p* > 0.05) (Supplementary Table S2). Our multilevel pleiotropy test indicated no level pleiotropy (*p* > 0.05) for the instrumental variables used (Supplementary Table S3). This suggests that the instrumental variables were causally related to PD via hypothyroidism, without confounding by other factors. The robustness of our findings was further confirmed in the leave-one-out sensitivity analysis (Supplementary Figure S2B). In addition, forest plots, scatter plots and funnel plots also confirmed the consistency of our MR analysis results (Supplementary Figures S3A–C).

## 4 Discussion

Our study shows that there is no common genetic variant between hypothyroidism and PD. Hypothyroidism does not have a causal effect on PD, but PD has a negative causal effect on hypothyroidism. This finding is inconsistent with previous studies showing that hypothyroidism increases the risk of developing PD (Charoenngam et al., 2022). A concurrent and prospective study using traditional statistics and novel machine learning models found that hypothyroidism is associated with an increased risk of PD (Gialluisi et al., 2023). *In vitro* and *in vivo* experiments have shown that thyroid hormones can prevent apoptosis of dopamine neurons (Kincaid, 2001; Lee et al., 2019; Menezes et al., 2019), suggesting a protective role of thyroid hormones on dopamine neurons in PD. However, hypothyroidism, characterised by insufficient thyroid hormone production, may exacerbate or promote the progression of PD. Recent bidirectional MR studies do not support a causal relationship between hypothyroidism and PD (Zeng et al., 2024). Our results are reliable and we used the most recent Finnish database with the largest sample size available for the study. First, in the co-localisation analysis of the two diseases did not find any common genetic variants were found, suggesting that variants in one disease do not increase the risk of the other. Second, despite the heterogeneity in the MR analysis of hypothyroidism on PD, the MR analysis overcame the confounding factors in traditional studies and ensured the reliability of causal inferences.

PD and hypothyroidism share similar pathogenic mechanisms, such as oxidative stress and inflammation. Studies have shown that apo D expression is upregulated in both diseases, and its antioxidant and anti-inflammatory effects provide an interesting avenue for further research (Fyfe-Desmarais et al., 2023). The mechanisms by which PD reduces the risk of hypothyroidism are unknown. We speculate that antioxidant mechanisms upregulated in PD may have a protective effect against the oxidative damage commonly seen in hypothyroidism. Future studies could investigate specific antioxidant levels and markers of oxidative stress in patients with PD compared to those with hypothyroidism. Altered immune-inflammatory pathways in PD may also play a protective role in the development of hypothyroidism. This hypothesis could be explored by examining cytokine levels and inflammatory markers in patients with PD versus those diagnosed with hypothyroidism to see if the reduced inflammatory response in PD is associated with a lower incidence of hypothyroidism. In addition, all current clinical pharmacological treatments can only alleviate the symptoms of PD, rather than slowing and curing the disease process. Investigating the role of oxidative stress and immune-inflammatory responses may provide new targets for PD treatment.

The motor dysfunctions associated with hypothyroidism may resemble those of PD, and given its treatability, accurate clinical diagnosis is important (Schneider et al., 2023). The high prevalence of PD and hypothyroidism in the elderly and of subclinical hypothyroidism in PD patients suggests a complex interaction between the two diseases (Munhoz et al., 2004). There is an association between thyroid hormones and PD, and most of the 12 SNPs may influence PD and thyroid function through immune mechanisms (Xu et al., 2022). The cardiovascular effects of hypothyroidism in PD and the improvement of fluctuating symptoms with thyroxine therapy in some patients further emphasise the need to assess thyroid function in the management of PD (García-Moreno and Chacón-Peña, 2003; Rodrigues et al., 2020). Although we did not find a common genetic variant between PD and hypothyroidism, the interaction between hypothyroidism and PD warrants further investigation.

Nevertheless, our study has limitations, including the heterogeneity of the instrumental variables in our MR analyses, which could be due to unobserved confounders. Also, as our GWAS data are predominantly from European populations, the applicability of our findings to other ethnic groups, such as Asians, remains uncertain. In addition, gender differences in the prevalence of hypothyroidism and PD (Chaker et al., 2017; Cerri et al., 2019), which were not accounted for in our analysis, may influence our results and should be a focus of future research.

In conclusion, our findings suggest that there is no shared genetic variation between hypothyroidism and PD, and interestingly, PD may have an inverse effect on the risk of developing hypothyroidism, a finding that may inform future research and clinical approaches.

## Data availability statement

Publicly available datasets were analyzed in this study. This data can be found at: https://www.finngen.fi/en/access_results.

## Author contributions

JLe: Writing – original draft. WH: Writing – original draft. YaL: Formal analysis, Writing – original draft. QZ: Investigation, Writing – review & editing. YiL: Formal analysis, Writing – original draft. QO: Investigation, Writing – original draft. XW: Formal analysis, Writing – review & editing. FL: Formal analysis, Writing – original draft. JLi: Investigation, Writing – review & editing. YX: Writing – review & editing.

## Footnotes

^1^ https://www.finngen.fi/en/access_results

^2^ https://github.com/chr1swallace/coloc

^3^ www.phenoscanner.medschl.cam.ac.uk
