# Supplementary material for: The potential protective role of Parkinson’s disease against hypothyroidism: co-localisation and bidirectional Mendelian randomization study
Source: Front Aging Neurosci. 2024 May 14;16:1377719. doi: 10.3389/fnagi.2024.1377719 (PMC11130391; doi:10.3389/fnagi.2024.1377719)
Supplement: Supplementary file 1 [file Data_Sheet_1.docx]

Supplementary Table 1: SNPs screened according to methodological criteria

| Exposure | SNP | effect_  allele | other_allele | beta | eaf | se | | pval | samplesize | F |
| --- | --- | --- | --- | --- | --- | --- | --- | --- | --- | --- |
| Hypothyroidism | rs10986433 | A | G | -0.234 | 0.721 | | 0.050 | 2.43E-06 | 350,652 | 22.224 |
|  | rs1178485 | T | A | -0.572 | 0.051 | | 0.122 | 2.90E-06 | 350,652 | 21.880 |
|  | rs13384101 | A | G | -0.344 | 0.171 | | 0.065 | 1.45E-07 | 350,652 | 27.652 |
|  | rs144776771 | C | T | 0.572 | 0.026 | | 0.122 | 2.54E-06 | 350,652 | 22.135 |
|  | rs17399904 | G | C | -0.746 | 0.035 | | 0.153 | 1.13E-06 | 350,652 | 23.699 |
|  | rs1912995 | G | A | 0.316 | 0.526 | | 0.0460 | 6.14E-12 | 350,652 | 47.284 |
|  | rs2476601 | G | A | -0.388 | 0.856 | | 0.0560 | 9.14E-11 | 350,652 | 41.997 |
|  | rs6925652 | C | T | 0.221 | 0.369 | | 0.0470 | 2.59E-06 | 350,652 | 22.102 |
|  | rs72818613 | T | G | -0.727 | 0.038 | | 0.148 | 8.51E-07 | 350,652 | 24.239 |
|  | rs9271364 | G | A | 0.277 | 0.425 | | 0.046 | 1.17E-09 | 350,652 | 37.017 |
|  | rs9611566 | T | C | -0.251 | 0.263 | | 0.054 | 3.43E-06 | 350,652 | 21.560 |
| PD | rs10271158 | G | A | 0.107 | -0.029 | | 0.023 | 3.18E-06 | 412,181 | 21.707 |
|  | rs114335056 | G | A | -0.148 | 0.183 | | 0.031 | 1.36E-06 | 412,181 | 23.339 |
|  | rs116661870 | T | C | 0.238 | -0.024 | | 0.049 | 1.50E-06 | 412,181 | 23.152 |
|  | rs117503845 | A | G | -1.346 | 0.448 | | 0.240 | 1.91E-08 | 412,181 | 31.580 |
|  | rs144174710 | G | A | -0.780 | 0.314 | | 0.161 | 1.64E-06 | 412,181 | 22.971 |
|  | rs151305702 | A | C | -0.140 | 0.014 | | 0.028 | 4.68E-07 | 412,181 | 25.390 |
|  | rs16854140 | T | C | -0.141 | -0.002 | | 0.030 | 3.06E-06 | 412,181 | 21.776 |
|  | rs186771067 | T | A | 0.252 | 0.049 | | 0.055 | 4.46E-06 | 412,181 | 21.054 |
|  | rs2509770 | A | T | -0.226 | 0.124 | | 0.049 | 3.08E-06 | 412,181 | 21.764 |
|  | rs2583990 | G | A | 0.153 | -0.013 | | 0.028 | 4.10E-08 | 412,181 | 30.101 |
|  | rs2732613 | C | A | -0.259 | 0.117 | | 0.041 | 3.69E-10 | 412,181 | 39.269 |
|  | rs34311866 | C | T | 0.125 | -0.105 | | 0.025 | 9.77E-07 | 412,181 | 23.974 |
|  | rs35603727 | A | G | 0.348 | -0.019 | | 0.049 | 1.87E-12 | 412,181 | 49.621 |
|  | rs3934591 | G | A | 0.106 | -0.080 | | 0.021 | 5.35E-07 | 412,181 | 25.134 |
|  | rs6982337 | G | T | 0.102 | -0.001 | | 0.021 | 1.15E-06 | 412,181 | 23.667 |
|  | rs73536723 | A | G | -0.460 | -0.223 | | 0.098 | 2.41E-06 | 412,181 | 22.235 |
|  | rs74963755 | C | A | 0.186 | 0.003 | | 0.040 | 4.44E-06 | 412,181 | 21.064 |
|  | rs75218231 | G | A | -0.730 | 0.165 | | 0.155 | 2.50E-06 | 412,181 | 22.168 |
|  | rs7650456 | G | C | 0.145 | 0.006 | | 0.031 | 4.80E-06 | 412,181 | 20.916 |
|  | rs77628790 | G | T | 0.231 | 0.022 | | 0.044 | 1.26E-07 | 412,181 | 27.934 |

Supplementary Table 2: Heterogeneity tests

| Exposure | Outcome | Method | Q | Q_df | Q_pval |
| --- | --- | --- | --- | --- | --- |
| Hypothyroidism | PD | IVW | 18.972 | 9 | 0.025 |
|  |  | MR-Egger | 19.034 | 10 | 0.040 |
| PD | Hypothyroidism | IVW | 15.629 | 18 | 0.618 |
|  |  | MR-Egger | 16.193 | 19 | 0.644 |

Supplementary Table 3: Pleiotropy tests

| Exposure | Outcome | MR-Egger Intercept | SE | P-value |
| --- | --- | --- | --- | --- |
| Hypothyroidism | PD | -0.006 | 0.034 | 0.869 |
| PD | Hypothyroidism | -0.024 | 0.032 | 0.462 |


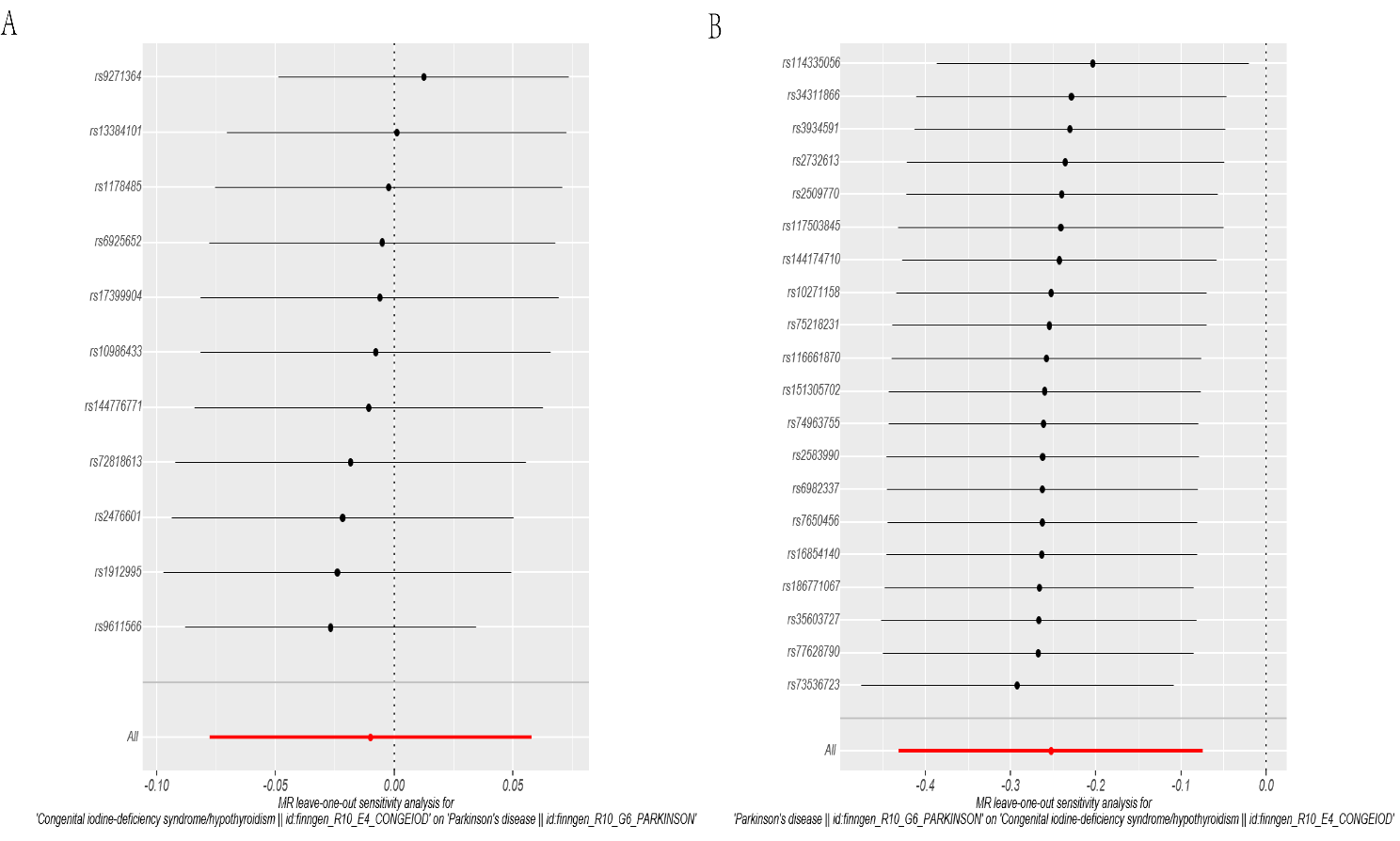


Supplementary Figure 1: Leave-one-out sensitivity analysis: Hypothyroidism on PD (A), PD on Hypothyroidism(B).


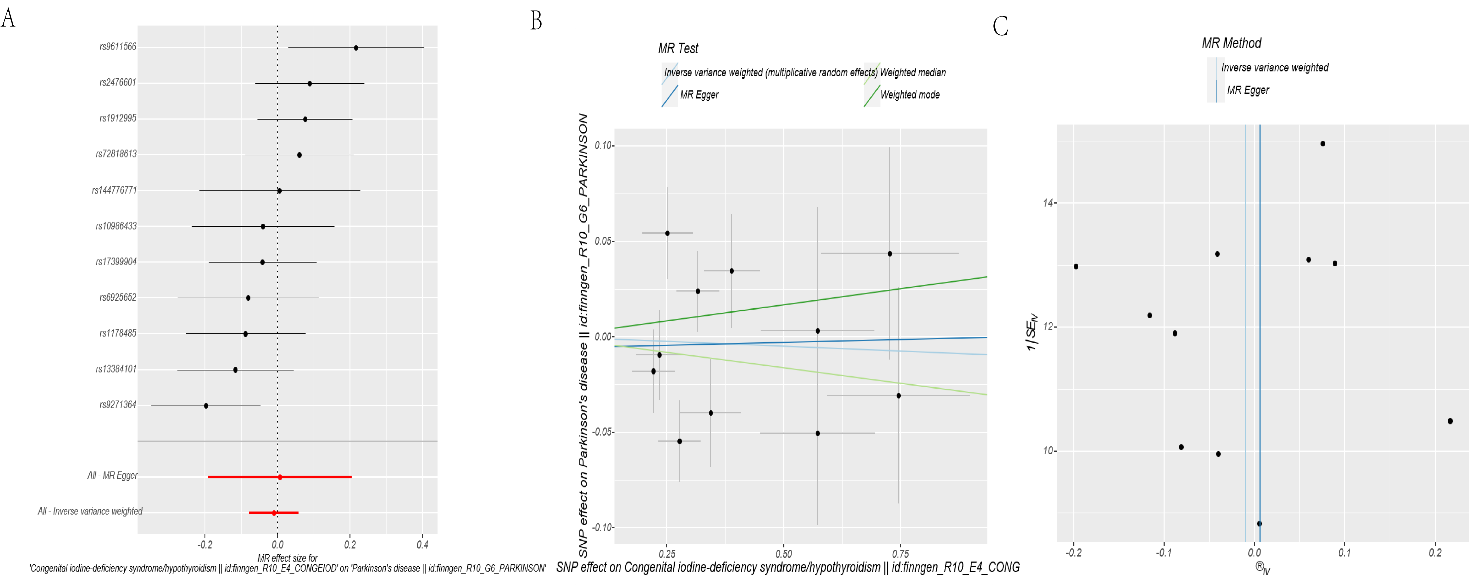


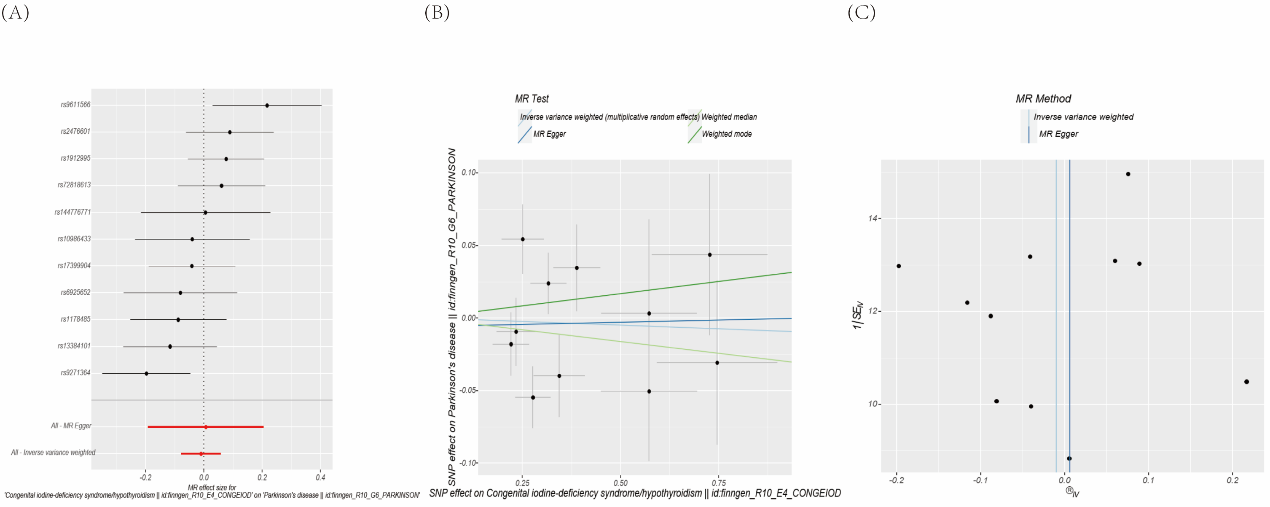


Supplementary Figure 2: Forest plot(A), Scatter plot(B) and Funnel plot (C) of the association of Hypothyroidism on PD.


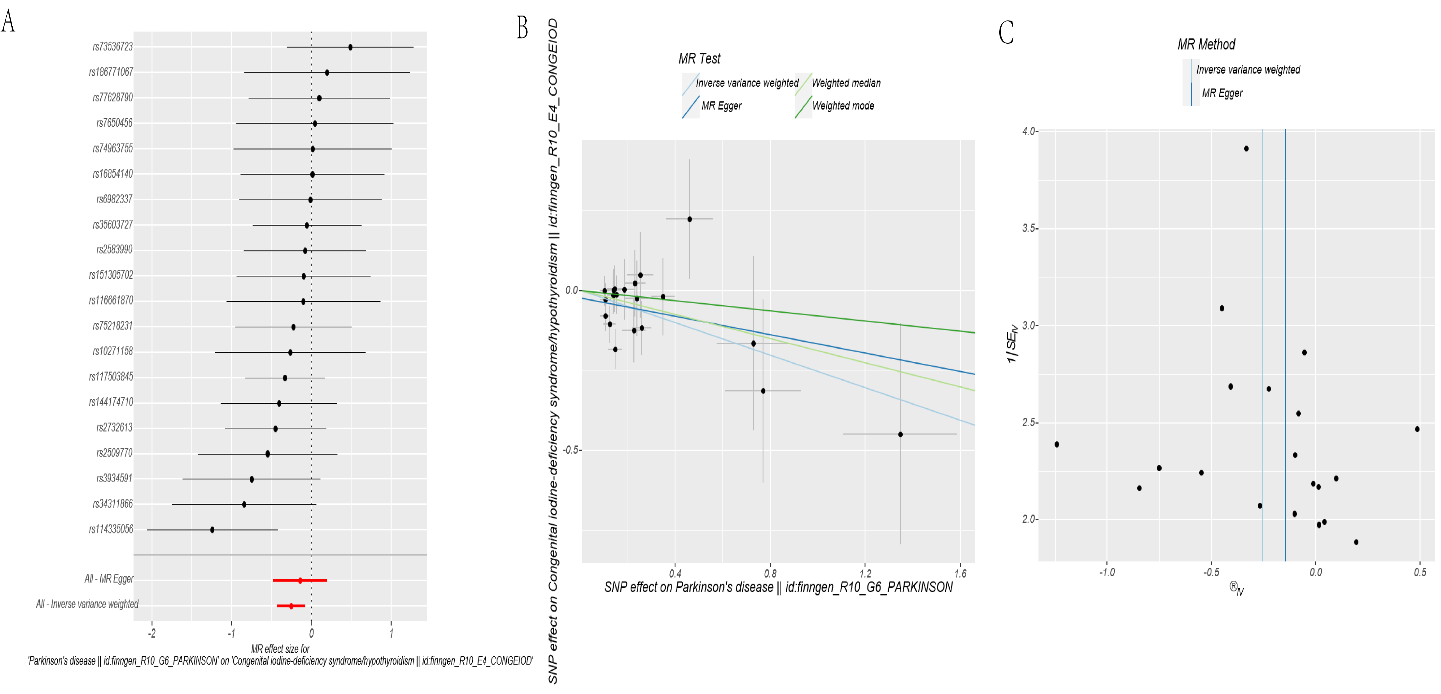


Supplementary Figure 3: Forest plot(A), Scatter plot(B) and Funnel plot (C) of the association of PD on Hypothyroidism.
